# Initial validation of a short version of the PERMA profiler in a national sample of rural veterans

**DOI:** 10.3389/fpubh.2024.1500659

**Published:** 2024-11-01

**Authors:** Emre Umucu, Teresa Ann Granger, Deyu Pan, Traci McGee, Eunae Han, Jim Yates, John Barnas, Crystal Barter, Beatrice Lee

**Affiliations:** ^1^Public Health Sciences, College of Health Sciences, The University of Texas at El Paso, El Paso, TX, United States; ^2^South Texas VA Medical Center, San Antonio, TX, United States; ^3^Department of Educational Studies in Psychology, Research Methodology, and Counseling, University of Alabama, Tuscaloosa, AL, United States; ^4^Research & Development, Tuscaloosa VA Medical Center, Tuscaloosa, AL, United States; ^5^Rehabilitation and Human Services, Penn State Wilkes-Barre, Lehman, PA, United States; ^6^Department of Counseling and Special Education, College of Education, The University of Texas, El Paso, TX, United States; ^7^Michigan Center for Rural Health, East Lansing, MI, United States; ^8^Department of Rehabilitation Sciences, College of Health Sciences, The University of Texas at El Paso, El Paso, TX, United States

**Keywords:** veterans, rural, service-connected disability, wellbeing, PERMA

## Abstract

**Background:**

Military veterans residing in rural areas face unique challenges that can impact their wellbeing, including limited access to healthcare resources, social isolation, and distinct environmental stressors. Despite growing interest in veteran wellbeing, there remains a gap in understanding how service-connected disabilities and health conditions intersect with wellbeing in rural contexts.

**Methods:**

This study employed a comprehensive approach to investigate the relationships between wellbeing, service-connected disabilities, and health outcomes among rural veterans. First, a short version of the PERMA Profiler was psychometrically validated for use among rural veterans. Then, associations between wellbeing and mental/physical health outcomes were examined. Lastly, differences in wellbeing between veterans with and without service-connected disabilities were evaluated.

**Results:**

The psychometric validation of the short-form PERMA Profiler yielded robust results, establishing its reliability and validity for assessing wellbeing among rural veterans. Significant positive associations were found between wellbeing and mental/physical health outcomes. Moreover, rural veterans with service-connected disabilities exhibited lower wellbeing scores compared to those without such disabilities.

**Conclusion:**

This study enhances our understanding of wellbeing among rural veterans, emphasizing the importance of considering service-connected disabilities and health conditions. The findings underscore the need for targeted interventions and support systems tailored to the specific needs of rural veterans, particularly those with service-connected disabilities. Recognizing and addressing these factors are crucial steps toward enhancing the overall wellbeing of this population.

## Introduction

U.S. Department of Veterans Affairs (1) estimated that 4.4 million veterans reside in rural communities, accounting for almost a quarter of all veterans in the United States. Rural veterans are more likely to be older, have more complex medical issues, have a service-connected disability, have lower incomes, and be unemployed when compared to their urban counterparts (1, 2). Rural veterans often face unique challenges, including limited access to healthcare resources, social isolation, and a distinct set of environmental stressors. As it stands, rural Americans also comprise a disproportionately large portion of veterans that have served across military branches (3). Further, rural veterans have been found to experience more health conditions and report significantly lower health-related quality of life in comparison to veterans that reside in urban areas (4, 5). Understanding the interplay between their wellbeing, service-connected disabilities, and health conditions is crucial for informing targeted interventions that address the specific needs of this population.

The wellbeing of military veterans has been a subject of growing interest, with a focus on factors such as mental health, social integration, and overall life satisfaction (6–12). Numerous studies have underscored the heightened risk of mental health, housing, and rehabilitation challenges among veterans, including conditions like depression, anxiety, suicidality, social isolation, and post-traumatic stress disorder (PTSD) (13–27), which would have negative effects on wellbeing (19, 28–30). The impact of these risk factors may be even more pronounced among rural veterans. Teich et al. (31) found that rural veterans were 70% less likely to receive mental health services and 64% less likely to access prescription medication when compared to urban veterans. As rural veterans are more likely to live farther from medical facilities, have fewer specialty care options and local providers, and encounter more transportation issues (32). These alarming differences and circumstances indicate an ongoing need to further examine the barriers that rural veterans are encountering. However, the complexities of wellbeing in the context of service-connected disabilities, particularly in rural settings, have received limited attention.

Rural areas pose unique contextual challenges that can impact the wellbeing of veterans. Limited access to healthcare services, geographical isolation, and a distinct rural culture may influence how veterans perceive and experience wellbeing (33–37). Furthermore, service-connected disabilities, which result from injuries or illnesses incurred or aggravated during military service, can have profound implications for overall wellbeing (11). Understanding the relationships between service-connected disabilities, health conditions, and wellbeing is essential for tailoring interventions to address the specific needs of rural veterans.

The PERMA Profiler, a 23-item self-report measure developed by Butler and Kern (38) based on Seligman’s (39) conceptualization of wellbeing, assesses the five pillars of global wellbeing – positive emotion, engagement, relationships, meaning, and accomplishment. The PERMA-Profiler has been widely used as a measure of wellbeing and has previously been validated in veterans (9, 40) and people with disabilities (29, 41). Yet, no study has been done to validate the PERMA Profiler for rural veterans. Additionally, Butler and Kern (38) suggested reporting the PERMA Profiler as a single score is a global indication of wellbeing, though omitting meaningful variation in different wellbeing domains. Given the limited healthcare resources in rural areas, particularly mental health resources, it is warranted to routinely administrate an efficient and reliable measure of wellbeing in medical settings for rural veterans. Validating a short version of the PERMA Profiler for veterans in the context of rural areas is a crucial step in enhancing our ability to understand and monitor their wellbeing.

The present study aims to add to current knowledge on wellbeing by validating a short version of the PERMA Profiler for rural veterans. We first conducted exploratory factor analysis (EFA) and cross-validated the EFA results using confirmatory factor analysis (CFA) on a separate sample to determine the optimal factorial structure of the short-form of PERMA Profiler. We calculated the coefficient alpha to provide reliability evidence. We then examined the relationship between wellbeing, service-connected disability, and health conditions among rural veterans, which has not received enough attention among rehabilitation researchers, providing additional validity evidence. By bridging gaps in the literature, this study provides valuable insights that can inform targeted interventions and support systems for enhancing the overall wellbeing of this unique population.

## Methods

### Procedure

The data for this study was collected from veterans living in rural settings upon ethics committee approval from the Institutional Review Board. Participants were eligible for our study if they met all of the following criteria: (a) a veteran who is 18 years old or older and (b) living in a rural area in the U.S. We used convenience sampling methods to collect our data. The survey was distributed by using media materials, including social media materials. We also reached out to our colleagues to help us collect data from their network. Data was collected using Qualtrics from December 2022 to March 2023. Data quality was checked utilizing attention check items (e.g., “Select correct responses: five plus 2 = seven.”; “Select the color option below: Car.”). Those who failed attention checks were removed from the dataset. We had a total of 1,022 veterans who initiated our survey. A total of 522 participants were removed from the dataset due to failing attention check items and not completing the survey, resulting in a total of 500 veterans living in rural locations.

### Participants

The mean age of participants was 34.86 (*SD* = 10.99). The majority of participants were male (*n* = 401; 80.2%). Most participants were non-Hispanic White (*n* = 357; 71.4%), followed by Black (*n* = 80; 16%), American Indian or Alaska Native (*n* = 29; 5.8%), Native Hawaiian or Pacific Islander (*n* = 14; 2.8%), Asian (*n* = 13; 2.6%), and others (*n* = 7; 1.4%). A total of 101 participants were identified as Hispanic (20.2%), and most participants had at least a high school degree (96.4%). Most participants were employed (*n* = 368; 73.6%). About 46% of participants reported having a service-connected disability. Regarding mental health conditions, participants reported depression (36.0%), anxiety (41.0%), PTSD (21.0%), bipolar disorder (15.4%), substance use disorders (6.4%), personality disorder (5.6%), and schizophrenia (4.0%). Regarding physical health conditions, participants reported migraine (20.6%), tinnitus (16.2%), paralysis (14.6%), hearing loss (14.0%), musculoskeletal disease (11.2%), Alzheimer’s disease (2.0%), and others (3.8%).

### Materials

We administered a demographic questionnaire to gather data about participants’ age, gender, race, and education. The PERMA-Profiler (38) is a 23-item scale measuring positive emotion, engagement, relationships, meaning, accomplishment, overall wellbeing, negative emotion, and physical health. Participants were prompted to rate each item (e.g., “To what extent do you feel loved?”) on an 11-point Likert scale from 0 (*never*) to 10 (*always*). In the current study, we selected a single item for each domain (i.e., positive emotion, engagement, relationships, meaning, and accomplishment) that had the highest factor loading in the original study (38), totaling five items and thus representing the short version of the PERMA Profiler. We also assessed participants’ service-connected disability status by using a single item (i.e., “Do you have a service-connected disability rating?”). Participants’ clinical status was measured using a single item (i.e., “Do you have any of the following conditions (Check all that apply)”). Participants were given multiple conditions such as depression, anxiety, hearing loss, and others. We also used a single item (i.e., “I’m always optimistic about my future”) from the Revised Life Orientation Test (LOT-R) (42) to measure optimism and a single item (i.e., “I tend to bounce back quickly after hard times”) from the Brief Resilience Scale to measure resilience (BRS) (43).

### Data analysis

For the first purpose of the study, a random split-half approach was adopted by performing exploratory factor analysis (EFA) on the first split-half data set (*n* = 247) and confirmatory factor analysis (CFA) on the second data set (*n* = 253). An EFA and a CFA were conducted with SPSS 28 and R, respectively. Nunnally and Bernstein (44) recommended having a minimum of 10 participants for each item in the instrument. In this study, a sample size of 247 for EFA and 253 for CFA were deemed sufficient for conducting factor analysis.

The internal consistency reliability coefficient (McDonald’s omega reliability) was computed to estimate the reliability of the short-form PERMA Profiler. A correlational analysis was conducted to provide concurrent validity evidence. For the second purpose of the study, a hierarchical multiple regression analysis was performed to more thoroughly investigate the relationships between wellbeing and overall health. For the last purpose of the study, an independent-sample *t*-test was conducted to compare the wellbeing scores of rural veterans with and without a service-connected disability. We used SPSS 29 (45) and Amos (45) for our analysis.

## Results

### Descriptive statistics

#### EFA

The 5 × 5 correlation matrix of the short-form PERMA Profiler was subjected to a factor analysis. The Kaiser-Meyer-Olkin (KMO) measure of sampling adequacy was 0.89 (>0.60), and Barlett’s test of sphericity was significant, *χ*^2^_(10, 243)_ = 733.78, *p* < 0.001, indicating suitability for factor analysis. Kaiser–Guttman’s “Eigenvalues greater than one” criterion and Cattell’s scree test (44) both indicated a one-factor measurement structure, accounting for 72% of the total variance. All items loaded significantly onto the general factor (ranging from 0.67 to 0.77; see Table 1).

**Table 1:** Factor matrix, communalities, means and standard deviation of items and total score, and reliability information.

Item	Factor loading	*h^2^*	*M*
P: How often do you feel positive?	0.85	0.73	5.25
E: To what extent do you feel excited and interested in things?	0.88	0.77	5.00
R: To what extent do you feel loved?	0.82	0.68	5.58
M: To what extent do you feel that what you do in your life is valuable and worthwhile?	0.86	0.74	5.54
A: How much of the time do you feel you are making progress towards accomplishing your goals?	0.84	0.70	5.40
Total *M* and *SD*	6.31 (1.83)		
Eigenvalue	3.61		
% Variance	72.25		
McDonald’s omega reliability	0.90		

P, positive emotion; E, engagement; R, relationships; M, meaning; A, accomplishment.

#### CFA

CFA is often used to cross-validate the factor structure of a psychological measure (46), allowing researchers to evaluate the fit between the postulated model and the observed data. Therefore, the one-factor structure of the short-form PERMA Profiler was estimated using a CFA with a second sample of rural veterans (*n* = 253). The model-data fit was examined using the chi-square goodness-of-fit test (*χ*^2^) as well as several alternative fit indices that are less affected by the sample size, including the *χ*^2^/*df*, the comparative fit index (CFI), the Turker-Lewis Index (TLI), the standardized root mean square residual (SRMR), and the root mean square error of approximation (RMSEA). To evaluate the overall goodness-of-fit criteria for the model, the *χ*^2^ should not be significant, *χ*^2^/*df* should be in the range of 1–3, the CFI and TLI should be equal to or greater than 0.95, SRMR should not exceed 0.05, and RMSEA should not exceed 0.08 (47, 48).

**Figure 1:**
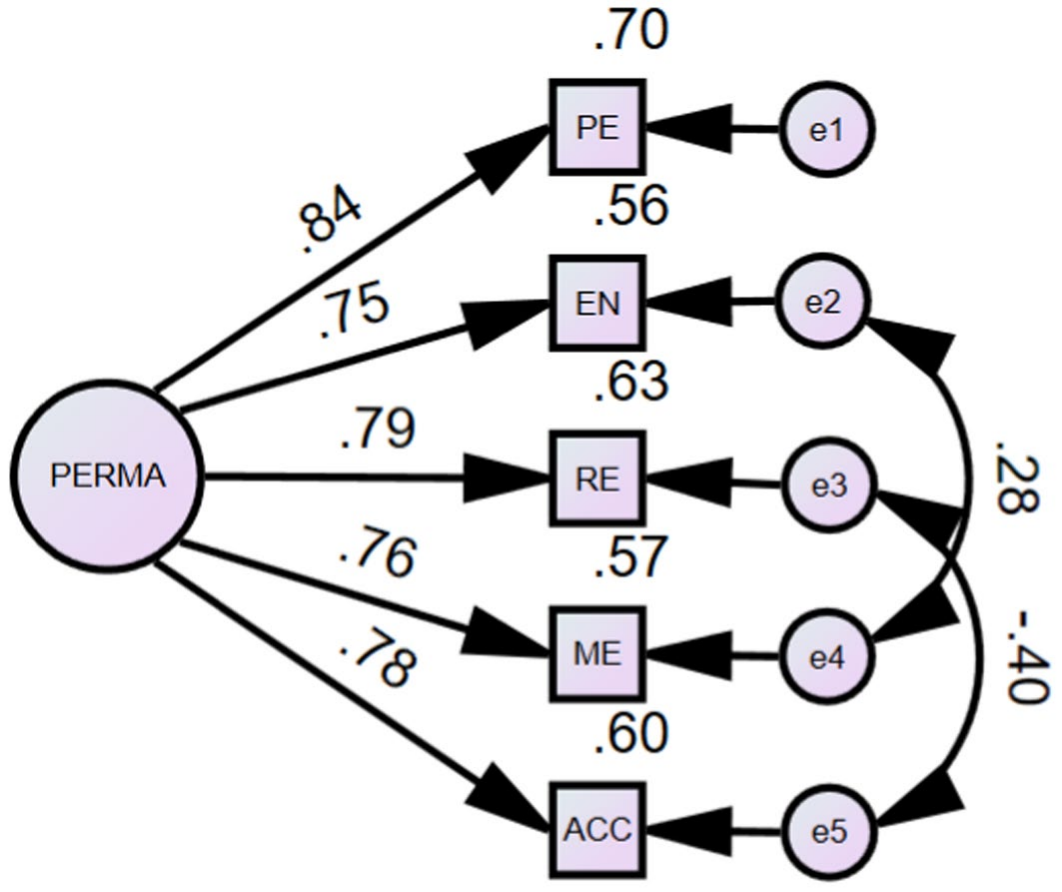
CFA results of the short-form PERMA-profiler.

The initial one-factor CFA model indicated a relatively poor fit for the data: *χ*^2^_(5, 253)_ = 28.96, *p* < 0.001, CFI = 0.96, TLI = 0.93, SRMR = 0.03, and RMSEA = 0.14, 90% confidence interval (CI) [0.09, 0.19]. However, an examination of the modification indexes indicated that two pairs of error terms should be correlated: Item *e2* (“To what extent do you feel excited and interested in things?”) with Item *e*4 (“To what extent do you feel that what you do in your life is valuable and worthwhile?”); and Item *e3* (“To what extent do you feel loved?”) with Item *e*5 (“How much of the time do you feel you are making progress towards accomplishing your goals?”). Given that these items are closely related to positive emotions, they may indeed influence one another. Therefore, it is theoretically justifiable to correlate these items. Results of the re-specified one-factor model indicated an excellent model fit: *χ*^2^_(3, 253)_ = 2.901, *p* = 0.41 is not significant, CFI and TLI are both 1.00, greater than 0.95, SRMR of 0.01 is less than 0.05, and RMSEA of 0.01 (90% CI [0.01, 0.11]) is below the value of 0.08. All these indexes meet the criteria of very good model fit (49). Factor loadings for the scale were significant (*p* < 0.01) ranging from 0.75 to 0.84. Figure 1 depicts the revised one-factor CFA model for the short-form PERMA Profiler.

#### Reliability

The internal consistency reliability coefficient for the short-form PERMA Profiler was computed to be 0.90, indicating excellent reliability in a sample of rural veterans.

#### Concurrent validity

The short-form PERMA Profiler was positively associated with other psychology constructs including optimism (*r* = 0.60, *p* < 0.001) and resilience (*r* = 0.41, *p* < 0.001).

#### Association between wellbeing and physical and mental health

A hierarchical multiple regression analysis was performed to more thoroughly evaluate the relationships between wellbeing and health outcomes. As seen in Table 2, after entering age, gender, and minority status, as control variables in the first step, PERMA uniquely accounted for a significant proportion of variance in overall health outcome (*R* = 0.53, *R*^2^ = 0.31, Δ*R*^2^ = 0.30, *F*(6, 499) = 44.56, *p* < 0.05). The standardized regression coefficients were significant for mental health (*β* = 0.35, *p* < 0.001) and physical health (*β* = 0.21, *p* < 0.001).

**Table 2:** Hierarchical multiple regression analysis predicting overall health outcome (*N* = 500).

Variable	*β*	*R^2^*	*ΔR^2^*	*F*
Step 1		0.01	0.01	1.69
Age	−0.11^+^			
Gender*^a^*	−0.02^+^			
Minority Status*^b^*	0.04^+^			
Education*^c^*	0.04^+^			
Step 2		0.31	0.30	44.56
PERMA	0.55*			

^a^Gender (female = 1); ^b^minority status (minority = 1); ^c^education (at least high school = 1). ^*^p < 0.05, ^+^*p* = n.s.

#### Association between wellbeing, service-connected disability, and health conditions

An independent-sample *t*-test was conducted to compare the wellbeing scores of rural veterans with and without a service-connected disability. Significant differences were found in the current study in wellbeing scores for rural veterans with a service-connected disability (*M* = 6.07, *SD* = 1.69) and rural veterans without a service-connected disability (*M* = 6.07, *SD* = 1.96); *t*(498) = 6.21, *d* = 0.24 *p* < 0.01. We also found that PERMA scores were negatively associated with number of mental health conditions (*r* = −0.32, *p* < 0.001) and number of physical health conditions (*r* = −0.16, *p* < 0.001).

## Discussion

The present study aimed to enhance the understanding of wellbeing among rural veterans by accurately and efficiently assessing wellbeing and investigating the relationships between wellbeing, service-connected disability, and physical and mental health. To achieve this goal, we conducted a psychometric validation of the short-form PERMA-Profiler, examined the associations between wellbeing and health outcomes, and evaluated differences in wellbeing among rural veterans with and without service-connected disabilities.

The psychometric validation of the short-form PERMA Profiler in a sample of rural veterans provides satisfactory reliability and validity evidence. The factor analysis results supported a one-factor measurement structure, accounting for a substantial proportion (72%) of the total variance. This suggests the short-form of PERMA-Profiler is a unidimensional measure of wellbeing in rural veterans. Thus, the findings suggest that the five pillars of wellbeing - positive emotions, engagement, relationships, meaning, and accomplishment - are interconnected and collectively contribute to the construct of wellbeing. This contrasts with previous research, such as Butler and Kern’s (38) identification of a five-factor structure, aligning more closely with Seligman’s (50) authentic happiness theory, which views happiness and wellbeing as a unified dimension. Additionally, our findings indicate that the short-form PERMA Profiler had the highest correlation with happiness (*r* = 0.75, *p* < 0.001), indicating a robust association between the two measures. The PERMA Profiler also demonstrates substantial correlations with optimism (*r* = 0.60, *p* < 0.001) and resilience (*r* = 0.41, *p* < 0.001), although these correlations are slightly lower compared to its correlation with happiness. Overall, these findings suggest that individuals who score higher on the short-form PERMA Profiler tend to report higher levels of optimism, resilience, and particularly happiness.

Considering that this study examined a population distinct from the participants in Butler and Kern’s^40^ research, the emergence of a one-factor structure may imply that rural veterans perceive, assess, or exhibit wellbeing measures, including the PERMA-Profiler, differently. This could also be attributed to the smaller number of items used compared to the original tool. Moreover, prior investigations yield varied results regarding the structure of the PERMA-Profiler. For instance, studies have proposed a five-factor model among Italian adults (51), a three-factor model among Malaysian adults (52), a two-factor model among Australian adults (53), as well as among student veterans in the United States (9). Additionally, a one-factor model was observed in young adult brain cancer survivors (29). These divergent findings underscore the complexity and potential variability in understanding wellbeing across different populations and contexts.

Despite the variance theoretical dimensionality of the measure, we found the measure exhibited good reliability and concurrent validity. The high internal consistency reliability coefficient (McDonald’s omega reliability = 0.90) further affirmed the scale’s reliability in this specific population. Additionally, concurrent validity was established through positive associations with related constructs such as optimism, resilience, and happiness. These findings contribute reliability evidence for the short-form PERMA Profiler in the context of rural veterans, reinforcing its utility as a tool for assessing wellbeing in this population.

Our results also demonstrated a significant positive association between wellbeing and both mental and physical health among rural veterans. The hierarchical multiple regression analysis revealed that, even after accounting for demographic factors, wellbeing uniquely explained a substantial proportion of variance in overall health outcomes. This underscores the importance of considering wellbeing as a distinct and influential factor in predicting health outcomes among rural veterans. The standardized regression coefficients indicated that wellbeing had a stronger association with mental health (*β* = 0.35) than physical health (*β* = 0.21), emphasizing the psychological dimension’s prominent role in overall health. Previous research has determined that rural veterans have worse health-related quality of life and are more likely to have physical health conditions, but not mental health conditions compared to their non-rural counterparts (5). More recent research supports this conclusion, finding rural veterans had lower mental health service use but not poorer mental health compared to non-rural veterans (54).

The study revealed notable differences in wellbeing scores between rural veterans with and without service-connected disabilities. Veterans with service-connected disabilities exhibited lower wellbeing scores compared to their counterparts without such disabilities. This finding suggests that service-connected disabilities may have a negative impact on the overall wellbeing of rural veterans. Moreover, the negative association between PERMA scores and the number of mental and physical health conditions further emphasizes the intricate relationship between wellbeing and health status. The observed negative correlation implies that a higher number of health conditions is associated with lower wellbeing among rural veterans.

This finding aligns with earlier studies indicating that disability correlates negatively with wellbeing (55–57). In line with prior research, Umucu et al. (6) observed that veterans with service-connected disabilities exhibited notably lower levels of wellbeing compared to those without such disabilities. Similarly, Bond et al. (28) noted that veterans with service-connected disabilities, when contrasted with established norms, reported lower life satisfaction, poorer mental health, increased symptoms of depression and posttraumatic stress disorder, as well as heightened financial distress.

## Limitations

The study has several limitations that impact the interpretation and generalizability of its findings. First, its cross-sectional design restricts the ability to establish causal relationships among variables, highlighting the need for longitudinal research. Second, sampling bias stemming from the use of a convenience sample of rural veterans may limit generalizability, as participants who opted in may differ from those who did not. Third, reliance on self-report measures, particularly for health conditions and disability status, introduces potential response bias and may not fully capture objective severity. Moreover, the predominantly male, non-Hispanic White participant demographic restricts generalizability to more diverse veteran populations, though such a demographic makeup is compatible with that of rural veterans. Social desirability bias and the simplistic measurement of service-connected disability further impact data reliability and completeness. Additionally, limited consideration of geographic variability, exclusion of certain health conditions, lack of contextual information, and removal of participants failing attention checks all contribute to gaps in understanding the complexities of wellbeing and health outcomes among rural veterans. Finally, we observed a significant attrition rate, with over half (51.08%) of participants excluded for failing attention checks. While excluding these participants helped enhance data quality, we acknowledge that this approach may inadvertently remove some individuals with cognitive challenges from the sample. This raises important considerations regarding the representativeness of our findings and highlights the need for careful consideration of how attention checks are implemented in future studies.

## Implications for practice and research

This study has several implications. First, rehabilitation and healthcare professionals who work closely with rural veterans may incorporate the short-form PERMA Profiler as a part of routine assessment. With only five items, the short-form PERMA Profiler can be easily administrated as a global indication of wellbeing to identify clients with greater needs. With satisfactory reliability and validity evidence for rural veterans, the short-form PERMA Profiler can also be used for treatment progress and outcome monitoring in the healthcare system. Specifically, with associations with services-connected disability and health outcomes, the short-form PERMA Profiler can inform tailored strategies to promote psychological wellbeing and mitigate the impact of health conditions. Additionally, with strong correlations with positive psychology constructs, such as happiness, resilience, and optimism, the short-form PERMA Profiler can be used by researchers to investigate the effectiveness of positive psychology interventions. Future research should examine the test–retest reliability of the short-form PERMA Profiler and explore the longitudinal dynamics of wellbeing in relation to service-connected disabilities and health outcomes, providing a more comprehensive understanding of the relationship between these factors.

## Conclusion

In conclusion, this study contributes to the understanding of wellbeing among rural veterans by examining the reliability and validity of the short-form PERMA-Profiler. We found evidence supporting a one-factor structure of the scale in this population. Our results also underscore the reliability and validity of the short-form PERMA-Profiler in assessing wellbeing among rural veterans. Importantly, our findings highlight the significant positive association between wellbeing and both mental and physical health, emphasizing the influential role of psychological factors in overall health outcomes. Additionally, differences in wellbeing scores between veterans with and without service-connected disabilities underscore the need for targeted interventions to support those facing additional challenges. However, several limitations, including sampling bias and reliance on self-report measures, must be considered when interpreting these findings. Moving forward, longitudinal research and tailored interventions can further elucidate the complex relationship between wellbeing, disability, and health outcomes among rural veterans, informing strategies to promote their psychological flourishing and overall wellbeing.

## Data Availability

The datasets presented in this article are not readily available because data is not available due to ethical considerations. Requests to access the datasets should be directed to EU, eumucu@utep.edu.
